# Successful treatment of gallbladder mixed adenoneuroendocrine carcinoma with neo-adjuvant chemotherapy

**DOI:** 10.1186/1746-1596-7-163

**Published:** 2012-11-27

**Authors:** Wu Song, Wenfang Chen, Sheng Zhang, Jianjun Peng, Yulong He

**Affiliations:** 1 Department of Surgery, the First Affiliated Hospital, Sun Yat-sen University, 58# Zhongshan Road 2, Guangzhou 510080, China; 2 Department of Pathology, the First Affiliated Hospital, Sun Yat-sen University, Guangzhou China

**Keywords:** Gallbladder, Neuroendocrine tumors, Neo-adjuvant chemotherapy, Mixed adenoneuroendocrine carcinoma

## Abstract

Mixed adenoneuroendocrine carcinoma (MANEC) carcinomas rarely occur in the gallbladder. Here we reported a case of giant gallbladder unresectable mass with local liver invasion and omentum metastasis, which proved to be neuroendocrine carcinoma (NEC) by biopsy, received successful radical operation after neo-adjuvant chemotherapy plus somatostatin treatment. The patient showed good response as the neoplasm diminished dramatically and showed clear margin after 6 courses of treatment. A radical operation including cholecystectomy, hepatic wedge resection of the gallbladder fossa segment and lymph node of group 8a and 8p resection was performed successfully. Postoperative histopathological examination revealed neuroendocrine carcinoma mixed with adenocarcinoma in the gallbladder wall. Followed up showed no evidence of recurrence after 7 months of the operation. We suggest that neo-adjuvant chemotherapy may be beneficial to gallbladder mixed neuroendocrine carcinomas in an advanced stage which could also be advantageous to NEC of other organs.

**Virtual slides:**

http://www.diagnosticpathology.diagnomx.eu/vs/2731892837743787

## Introduction

Neuroendocrine tumors are uncommon diseases which mostly occur in the gastrointestinal tract, pancreas and lung. Epidemiology data from several medical centers in western countries indicate the incidence of NETs is about 2.5-5 /100,000 people. NETs occur in the gallbladder are rather rare and only account for 0.5% of all NETs [1]. Recently, probably due to the progress of the diagnostic techniques and the rising awareness of this uncommon tumor, the incidence of the NETs has been increasing [1-3] as well as the gallbladder NETs [4]. Additionally, Because of the aggressiveness of the tumor and lack of specific symptoms, patients are often diagnosed at an advanced stage when radical surgery is not available. Until now no consensus was reached in terms of the standard treatment, chemotherapy strategy and operational procedure for gallbladder NETs. Here, we report a rare case diagnosed as unresectable gallbladder neuroendocrine carcinoma (GB-NEC) by preoperative biopsy, who successfully obtained radical resection after 6 periods of neo-adjuvant chemotherapy and somatostatin treatment, finally was proved to be mixed adenoneuroendocrine carcinoma (MANEC).

## Case report

### Clinic data

**Figure 1:**
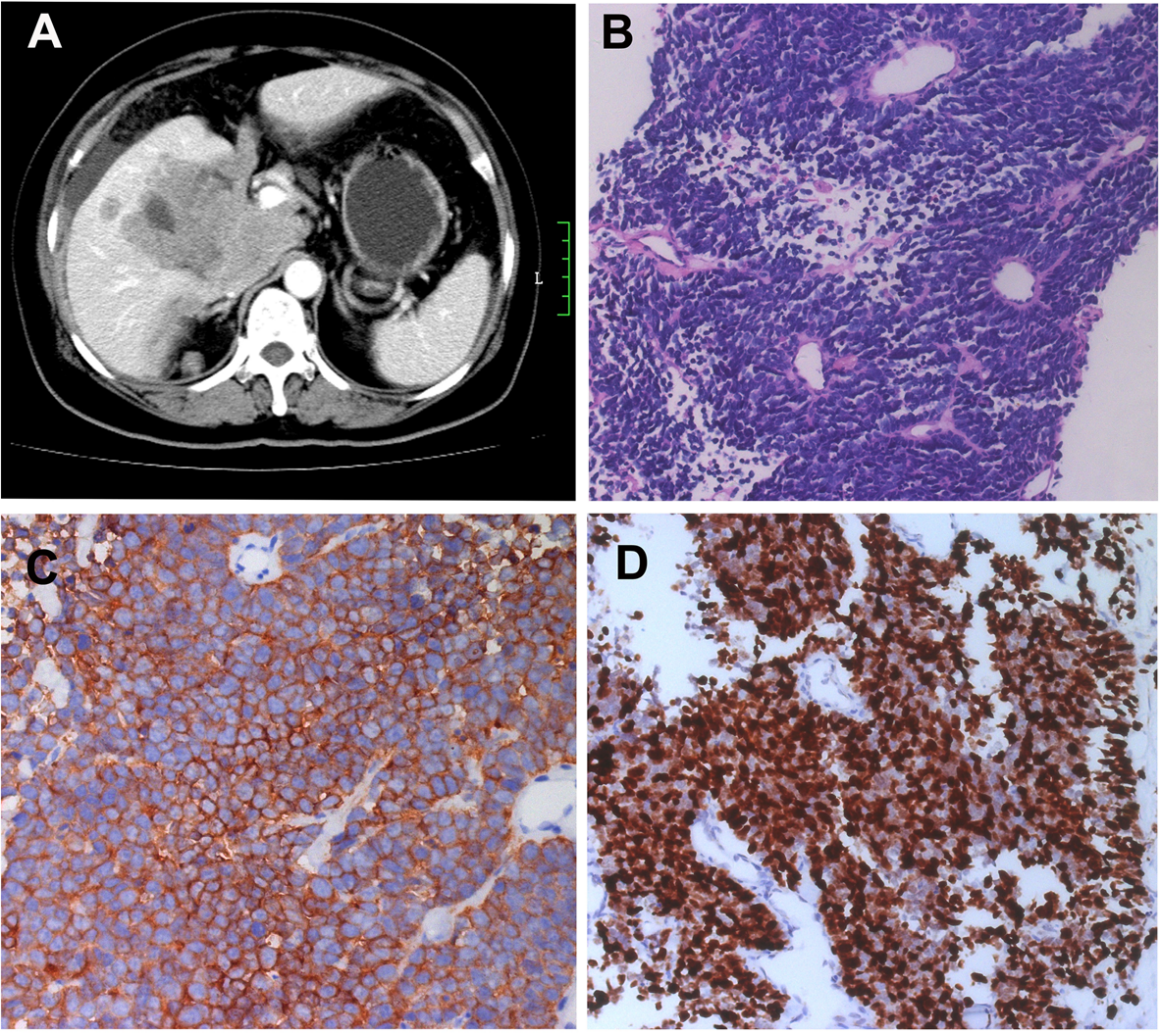
**Radiological and pathological data of the case before chemotherapy. A**. CT scan showed a gallbladder mass invading liver, head of pancreas with metastasis of peritoneum and enlarged lymphnodes in hepatic hilar. **B**. Diffuse small round cells with scanty cytoplasm and round nucleus (HE×200). **C**. Strong positive cytoplasmic expression of Syn (IHC×400). **D**. Over 80 % tumor cells show positive expression of Ki-67 (IHC×200).

A 55-year-old woman complained of mild epigastric discomfort was found to have a large impalpable mass in the gallbladder area with ultrasound examination. She was then admitted to the first affiliated hospital of Sun Yat-sen University. She had history of chronic cholecystitis for five years and no operation or other chronic diseases were found. Physical examination was negative. A slight impairment of liver function was found with AST 57 unit/L and ALT 68 unit/L (normal range<40) while albumin and bilirubin were within the normal range. Blood and urine analysis were normal. Her serum tumor markers CEA was 43 ug/L (normal range 0~5 ug/L), CA 125 465 U/ml (normal range 0~35 U/ml) and CA 19–9 100 U/ml (normal range 0~35 U/ml), all of which showed a significant elevation. Blood hormone test showed her serum CgA increased to 220 ng/ml (normal range17~34 U/L), while 5-HIAA, 5-HT and cortisol level were normal. Abdominal ultrasound examination revealed massive ascites together with a mass measuring 14 cm×8.4 cm in the gallbladder bed area closely adhering to the pancreatic head and liver. CT scan suggested that the gallbladder neoplasm invaded the neighboring liver, pancreatic head, peritoneum and omentum (Figure 1A). Lymph nodes enlargements were found in hepatic hilar region and liver segment 5 metastasis was suspected. PET/CT scan also demonstrated the results, without any other distant hyper-metabolic foci.

### Preoperational pathological findings

Diagnostic biopsy was thereafter performed. Histopathologically, the neoplasm was composed of diffuse small cells with scanty cytoplasm. The nuclei were round or short spindle-shaped exhibiting finely granular nuclear chromatin and inconspicuous nucleoli with large amount of mitotic count (>20/10HPF). Tumor cells tend to be located around the small vessels in some area forming pseudorosettes (Figure 1B). Immunohistochemistry staining revealed diffuse strong expression of CD56, CgA, NSE and Syn (Figure 1C). Ki-67 index was over 80% (Figure 1D). The panel of antibodies which reviewed diffuse strong expression were summarized in Table 1. Epithelial marker CK was positive while other markers such as CK7, CK20, and EMA were negative. Other markers such as LCA, CD99, Vimentin and S-100 were all negative. Gene detection showed no gene disruption in EWSR1 gene locus. Therefore, small cell neuroendocrine carcinoma in the gallbladder was diagnosed.

**Table 1:** The panel of immunohistochemical reagents which showed diffuse positive

**Antibody**	**Clone**	**Manufacturer**	**Dilution**
CK	AE1/AE3	Dako	1/200
CD56	CD564	Novocastra	Ready to use
CgA	5H7	Novocastra	Ready to use
NSE	E27	ZSGB-BIO	Ready to use
Syn	27 G12	Novocastra	Ready to use
Ki-67	SP6	ZSGB-BIO	1/400

### Treatment and following-up

**Figure 2:**
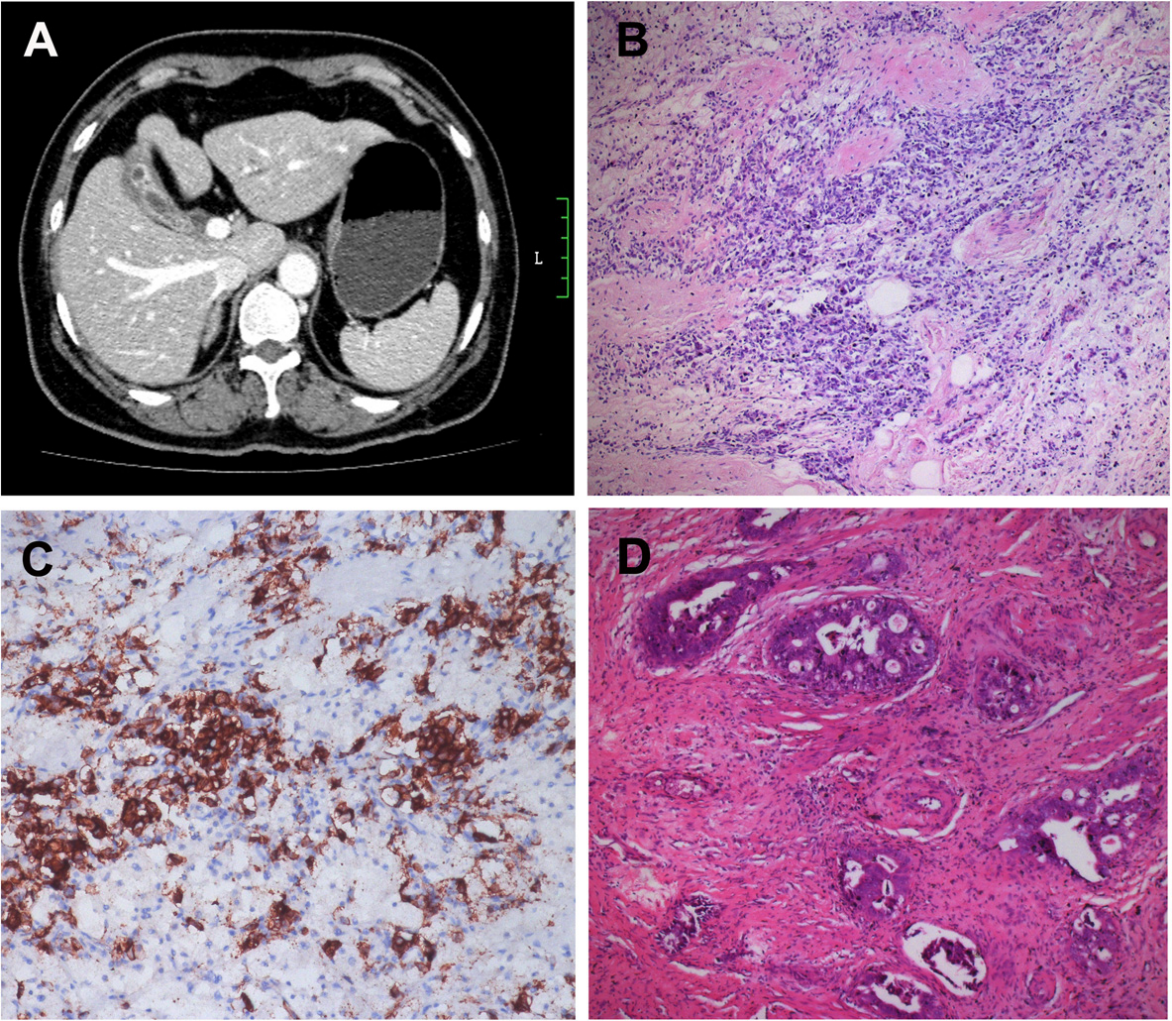
**Radiological and pathological data of the case after chemotherapy. A**. The mass decreased and was limited to the gallbladder fossa with clear margin. **B**. Small round-shaped tumor cells in gallbladder wall with marked interstitial fibrosis (HE×200). **C**. Positive expression of CgA in the cytoplasma of the tumor cells (IHC×200). **D**. Scattered large and cribriform glands infiltrate the mucous and muscularis layers (HE×200).

Considering the tumor is unresectable at this stage, neo-adjuvant chemotherapy and somatostatin treatments were adopted based on multi-disciplinary team (MDT) based discussion. The chemotherapy regiment is combination of Carboplatin 300 mg/m^2^, VP16 100 mg/m^2^, Paclitaxel 180 mg/m^2^ combined with Octreotide 30 mg/month. The patient showed good response to the chemotherapy and the abdominal ascites was relieved after two courses of treatment. She presented mild anorexia and hair loss as the side effects of chemotherapy, which was alleviated after the supportive and nutritional therapy. After 4 courses of treatment, her serum tumor markers including CEA, CA125 and CA19-9 decreased to normal range. CT scan suggested the size of the mass reduced dramatically and the enlarged lymph node in hepatic hilar region can not be detected any more. Liver lesion in the fifth segment was found to be not metastasis but a hepatic cyst. PET/CT scan indicated a hyper-metabolic nodule in the gallbladder area, with no other lesion all around the body. After 6 courses of treatment, her serum tumor markers remain normal and CT scan found that the tumor was limited to the gallbladder area with clear margin to the pancreatic head (Figure 2A). Two enlarged lymph nodes were found in the hepatodudenal ligament. Considering the satisfying effect of the neo-adjuvant chemotherapy, we decided to perform radical operation on this patient based on MDT discussion.

Intra-operative findings showed a hard mass measuring 7 cm×3 cm×2 cm in the gallbladder fossa, with clear margin to the pancreas. Palpable lymph nodes were found in the hepatodudenal ligament (group 12B). No metastasis was found in the liver and no para-aortic lymph node enlargement was detected. Cholecystectomy, hepatic wedge resection of the gallbladder fossa segment, lymph node dissection in hepatodudenal ligament and common hepatic artery (group 8a and 8p) were then performed. Postoperative pathological findings showed diffuse small round-shaped tumor cells in the gallbladder wall with marked interstitial fibrosis (Figure 2B). Compared with the biopsy, the tumor cells’ size, cell density and nuclear mitosis decreased. Immunohistochemistry staining revealed positive expression of CD56, CgA (Figure 2C), NSE, Syn and CK. The Ki-67 index is over 20%. In addition, scattered large, irregular and cribriform glandular composed of columnar neoplastic cells were found in the mucosa and muscularis layers of the gallbladder wall (Figure 2D). This moderately differentiated adenocarcinoma consisted about 40 percents of the whole tumor. Gallbladder serosa was not penetrated by the dysplasia glandular cells. There was no lymphovascular invasion in the sections and no metastasis was found in the dissected lymph nodes. The patient was diagnosed as MANEC (T3N0M0, stage III A).

Postoperative adjuvant chemotherapy and somatostatin treatment were continued for 3 more courses. A regularly followed up showed tumor biomarkers remain in normal range and CT scan found no evidence of recurrence after 7 months of the operation.

## Discussion

There are considerable discrepancies about the predilection sites of the NETs according to the database of various countries which may due to the different race [1,5]. Though gastroenteropancreatic NETs are the most common type in the NETs representing 65-75% of all cases [6], they also occur in other organs such as ovary, testis and hepatobiliary system and so on. Gallbladder neuroendocrine tumors (GB-NETs) are extremely rare which only represent 2% in all gallbladder tumors [1].

The average onset age of GB-NETs is 64 years old with a female preponderance [7] , which is very similar with primary colorectal NEC that is reported to have a median age of 60 years and a female to male ratio of 2:1 [8]. NETs are divided into functional and nonfunctional depending on whether the tumor cells can produce hormones and induce endocrine syndromes. Up to now 13 types of neuroendocrine cells have been found, which could release different types of bioactive molecules [6]. Most of the primary colorectal NEC patients have no carcinoid syndrome, so as the majority of the GB-NETs leading to it’s easily to be omitted in the earlier stage [8-10]. Patients often complain with nonspecific symptoms such as abdominal pain, epigastric discomfort and jaundice, with only 3.3%-3.7% in all cases presenting hormone-related syndromes [11]. As in our case, the patient complained about mild epigastric discomfort, without any hormone-related clinical manifestation.

Gallbladder carcinomas are believed to originate from malignant transformation of epithelial atypical hyperplasia which often occurs during the chronic inflammation [12]. The origin of GB-NETs remains uncertain because neuroendocrine cells do not naturally exist in the mucosa of the gallbladder. One of the theories proposed that neuroendocrine cells could be generated from the stem cells deviating from their original differentiation during metaplastic process especially intestinal type [13]. As reported by Sakamoto [14], 10.7% of intestinal metaplasia was found in 103 cholecystolithiasis cases who had received cholecystectomy. Cholecystolithiasis and cholecystitis were also found in most cases of GB-NETs, which suggested GB-NETs might relate to chronic inflammation of the gallbladder [15] , while only two of ten cases of colorectal NEC have related inflammatory diseases such as ulcerative colitis and Crohn's disease. This difference may indicate that the origination and mechanism of GB-NETs is quite different from colorectal NEC [8]. As in our case, the patient also had history of chronic cholecystitis. Proliferation and aggregation of these cells in the metaplastic epithelium could be an early sign for the gallbladder neuroendocrine tumors. Very few cases of concurrent adenocarcinoma and neuroendocrine tumor in the gallbladder were reported [16-18]. In our case, both glandular and neuroendocrine differentiations were observed and no transitional region between the two parts was found, which suggested the tumor might arise independently from two different precursor cells in a synchronous fashion or arise from a multipotent stem cell. Another theory postulate heterotopic pancreas in the gallbladder may play a role in origin of GB-NETs. The enzyme secreted by the heterotopic pancreas would damage the gallbladder mucosa and lead to metaplasia, which was believed as a possible etiology for GB-NETs [19,20].

The diagnosis of GB-NETs is rarely made preoperatively because there is no specific symptoms and imageological change. Ultrasound, CT scan, MRI, PET-CT and somatostatin receptor scintigraphy (SRS) can provide useful information of NETs, but confirmed diagnosis can only be made by pathological examination. Endoscopic ultrasonography with fine needle aspiration can increase the diagnostic sensitivity from 74% to 90%, compare to the endoscopic ultrasonography only [21]. Definite diagnosis relies on diffuse and intensive expression of marker proteins such as chromogranin A (CgA), neuron-Specific Enolase (NSE) and synaptophysin (Syn). Because of the extremely rareness of the primary NETs of the gallbladder, metastasis from other organs such as lung should be excluded carefully [22]. Recent grading system suggested by WHO and ENETS depend mainly on Ki-67 index and mitotic count [23]. Mixed adenoneuroendocrine carcinoma is diagnosed only when both portions are more than 30% in the pathological examination. In our case, adenocarcinoma was not detected in the biopsy but in the surgical specimen. We believe that it’s because of the specimen taken by the biopsy is limited so that the adenocarcinoma was omitted unintentionally. Another reason might be the curative effect of neo-adjuvant chemotherapy and somatostatin treatment, which killed majority of the tumor neuroendocrine cells and made the NEC portion less predominant.

Personalized and comprehensive therapy is preferred in treatment of NETs. Surgery, radiotherapy, peptide receptor mediated radionuclide therapy, chemotherapy and biological target treatment are all optional in NETs. Different surgical procedures such as mere cholecystectomy and cholecystectomy plus liver segment resection and lymph node dissections were reported according to different cases [24]. It is estimated that 74% of the GB-NETs patients will suffer from recurrence and metastasis after mere cholecystectomy [25]. Laparoscopic cholecystectomy is not preferred because it may cause intraperitoneal dissemination of the GB-NETs [26,27]. For early stage of GB-NETs such as T1N0, cholecystectomy is suggested. For advanced stage, cholecystectomy plus liver segment resection and lymph node dissection could increase the 5-year overall survival rate [21]. In some cases, chemotherapy was reported to increase the median survival time (4-31 months) and relief the symptoms in GB-NETs [28]. The objective response rate (ORR) of EP regimen in GEP-NETs is 53-67%, while its median survival time is shorter than 16 months [29]. Somatostatin analogues can inhibit the secretion of a broad range of hormones by binding to the somatostatin receptors on the NETs cell membrane. Recently, studies also found that somatostatin analogues can inhibit tumor cell growth directly by modulating the signal transduction of proliferation and apoptosis. Long-acting octreotide or lanreotide could prolong the overall survival rate of in metastatic mid-gut NET patient [30].

The prognosis of GB-NETs is related to the tumor size, invasion depth, differentiation degree and metastasis state, with a 60.4% 5-year overall survival rate [15]. Poor differentiation and accompanied with adenocarcinoma predict a worse prognosis, while tumor limited to the gallbladder wall is a good prognostic indicator. For GB-NETs with local invasion and lymph node metastasis, patients who received extended lymph node dissection would still suffer from recurrence and metastasis and the median survival time is only 30.3 months [25]. Extended operation including cholecystectomy, liver segment resection and lymph node dissection could increase the 5-year survival rate from 21.3% to 60.4% [31]. NCCN guideline indicated patients with the unresectable neuroendocrine tumor could receive palliative operation, adjuvant chemotherapy and somatostatin analogue treatment [32]. In our case, neo-adjuvant chemotherapy and somatostatin treatment successfully transformed the unresectable neoplasm and made radical operation available. Although at this stage it is hard to judge whether the radical operation could bring any long term advantage to patient of this kind, it does provide valuable experience in the management strategy of advanced gallbladder neuroendocrine tumors.

## Conclusion

GB-NECs are rare malignancies with aggressive biological behavior. Most of the cases are non hormone-producing and often asymptomatic leading to missed diagnosis in early stages. Preoperational pathological diagnosis and identification of clinical stage are of vital importance. Neo-adjuvant chemotherapy and somatostatin treatment got satisfying effects in this unresectable case which could also be instructive in NEC of other places. We suggest that for those NETs of advanced stage, patients may benefit from preoperative pathological diagnosis and standard neo-adjuvant chemotherapy which may also make complete removal of the tumor by surgery possible.

### Consent

A written informed consent was obtained from the patient for publication of this case report and any accompanying images. A copy of the written consent is available for review by the Editor-in-Chief of this journal.

## Abbreviations

MANEC: mixed adenoneuroendocrine carcinoma; NEC: neuroendocrine carcinoma; NET: neuroendocrine tumors; GB-NEC: gallbladder neuroendocrine carcinoma; EWSR1: Ewing sarcoma breakpoint region 1; SRS: somatostatin receptor scintigraphy; EP: VP16 and cis-platinum.

## Competing interests

The authors declare that they have no competing interests.

## Author’ contributions

WS and WFC contributed equally to this work. WS drafted the paper; WFC made analysis of the pathological examination and revised the paper carefully; SZ made contributions to acquisition of clinical data; JJP carried out the immunohistochemical staining; YLH designed and guided the research. All authors read and approved the final manuscript.
